# Benjamin H. Catching

**Published:** 1900-02

**Authors:** 


### Obituary.

### BENJAMIN H. CATCHING, D. D. S.

His many friends and the dental profession at large will learn with great regret of the death of Dr. Catching, of Atlanta, Ga., so well known through his numerous contributions to dental literature. For many years he has suffered from attacks of nervous exhaustion, the result of too ardent a devotion to work in the many spheres of activity in which his energies were expended. No one thought, however, and probably he least of all, that under the strain the end of all labor for him would come so soon. It was early in the morning of November 23d, 1899, just as he was about to leave his home to enter upon the duties of the day, that the stroke came, and within five minutes of the apopletic seizure all was over. "The silver cord was loosed and the golden bowl broken."

Benjamin Holliday Catching was, at the time of his death, fifty-one years of age. He was born June 28th, 1848, at Georgetown, Miss., of an ancestry distinguished for honorable service in the history of the State, his great-grand-father, Hon. Benjamin Catching, having taken an active part as a delegate in the convention which framed the original State Constitution.

Under the preceptorship of Dr. J. S. Knapp, of New Orleans, La., he began his study of dentistry, and later matriculated in the Baltimore Dental College, from which institution he graduated with distinction, and as valedictorian of his class, in March, 1870.

In the autumn of 1871 he entered upon active practice in Canton, Miss., where he remained until 1881, at which time, attractee by the superior opportunities as a field for practice offered by Atlanta, Ga., which had just entered upon its epoch of almost unprecedented growth and prosperity, he removed to that city, and there remained up to the time of his deaih.

Although he secured a large practice his predilection for literary pursuits soon led him into the field of journalism. He was the founder, and for eight years the editor of the "Southern Dental Journal." On retiring from that position he began the publication of "Catching's Compendium of Practical Dentistry," which furnished annually during the five consecutive years of its publication a most valuable compilation of all the important contributions to dental art and science made in this and foreign countries. It was by this work that he was best known, and it undoubtedly constituted his most important service to his profession. After the appearance of the fifth volume, 1896, ill-health from overwork compelled the discontinuance of its publication; and, with the exception of an occasional contribution to professional journals, he desisted from all literary labor until 1897, when he established the "American Dental Weekly." But, even with the aid of five colaborators, the labor involved in this publication was so enermous that the task proved beyond his strength, and with the issue of the fifty-second consecutive number the journal was discontinued.

He was ex-President of the Southern Dental Association and a member of the American Dental Association, which two organizations are now merged in the National Dental Association. He was also a member of the Georgia State Dental Faculty, and for four years served as a member of the Georgia State Board of Dental Examiners.

Dr. Catching was deeply religious in his convictions, and an active member of the Methodist Church, He was upright in all the relations of life, a good citizen, a devoted husband, a kind father, a Christian gentleman.

He was married June 15th, 1870, to Miss Mattie Sanders, of Georgetown, Miss., who, with a son and three daughters, survives him.—*Dental Brief.*

